# Multiscale Horizontal Visibility Graph Analysis of Higher-Order Moments for Estimating Statistical Dependency

**DOI:** 10.3390/e21101008

**Published:** 2019-10-16

**Authors:** Keqiang Dong, Haowei Che, Zhi Zou

**Affiliations:** College of Science, Civil Aviation University of China, Tianjin 300300, China; caucdong@126.com (H.C.); zouzhi1216@126.com (Z.Z.)

**Keywords:** information exchange, horizontal visibility graph, statistical moment, interaction

## Abstract

The horizontal visibility graph is not only a powerful tool for the analysis of complex systems, but also a promising way to analyze time series. In this paper, we present an approach to measure the nonlinear interactions between a non-stationary time series based on the horizontal visibility graph. We describe how a horizontal visibility graph may be calculated based on second-order and third-order statistical moments. We compare the new methods with the first-order measure, and then give examples including stock markets and aero-engine performance parameters. These analyses suggest that measures derived from the horizontal visibility graph may be of particular relevance to the growing interest in quantifying the information exchange between time series.

## 1. Introduction

Time series correlation analysis has been widely used in real-world systems including numerous components with greatly nonlinear interactions to detect the correlation among individual components. In recent years, the problem of correlation analysis has also been transformed to complex network analysis [1,2,3,4,5]. Among these, the visibility graphs (VGs) and horizontal visibility graphs (HVGs) have been proposed. 

The VG, which converts a time series into a graph, inherits several properties of the time series in its structure [6]. Consequently, fractal series are converted into scale-free networks, enhancing the fact that power law degree distribution is related to fractality, something highly discussed recently [6,7].

The HVG, to simplify calculating, has been introduced and explored in the context of graph-theoretical time series analysis [8]. By extracting topological features from the associated HVGs, which have shown to be informative on the class of dynamics, HVGs have been applied to characterize empirical signals [9]. Moreover, many statistical properties of the HVGs can be theoretically derived [10].

Quite a few topological properties of VGs and HVGs mapped from the time series of different real complex systems including financial markets [11,12,13], biological systems [14,15,16,17], ecological systems [18,19], and some other complex systems [20,21] have been studied numerically and analytically.

Furthermore, in many real-world applications, complex systems controlled by regulatory mechanisms that operate on different time scales, generate time series that exhibit highly variable fluctuations at multiple levels of resolution [22,23,24,25]. In order to investigate the multiscale characteristic, Gao et al. proposed a multiscale limited penetrable HVG for analyzing a single nonlinear time series and constructed a multiscale complex network from multivariate time series [5,26]. Zhang and Shang introduced a multiscale HVG analysis to measure time irreversibility based on a phase-space reconstruction method [27]. Li and Zhao proposed a multiscale horizontal visibility graph correlation analysis for quantifying the intrinsic interactions between two non-stationary time series [10]. Zhang et al. employed the multiscale horizontal visibility graph similarity to identify the interaction in an aero-engine gas path system [28].

In construction, the proposed multiscale methods do not make any use of higher-order statistics such as the higher-order moments, so they do not commonly embody variance and skewness properties of the signal [5,10,26,27,28]. In order to improve the detection and estimation performance for quantifying the intrinsic interactions between two non-stationary time series, this paper incorporates the higher-order moments with the multiscale horizontal visibility graph, and proposes the methods of second-order multiscale horizontal visibility graph correlation analysis (2nd-order MHVGCA) and third-order multiscale horizontal visibility graph correlation analysis (3rd-order MHVGCA). Things change rapidly in dynamic systems, and the system’s situation can deviate from the mean at different time scales. Thus, the 2nd-order MHVGCA and 3rd-order MHVGCA applied for high-frequency dynamical systems analysis can be further explored. We compared the higher-order MHVGCA with other measures, and then applied it to a stock time series and aero-engine time series.

The rest of the paper is organized as follows. In the Section 2, the traditional MHVGCA methodology is reviewed and the 2nd-order MHVGCA and 3rd-order MHVGCA are proposed. The proposed methods and their comparison with the conventional method are described in Section 3. The proposed methods are further applied to analyze a stock time series and aero-engine time series in Section 4. Finally, some conclusions are drawn in Section 5.

## 2. Methodologies

### 2.1. Multiscale Horizontal Visibility Graph Correlation Analysis Method

The multiscale horizontal visibility graph correlation analysis (MHVGCA) was introduced in [10,28] in order to catch any intrinsic interactions between two non-stationary time series. Let lowercase letters, $\left\{x_{t},t=1,2,\ldots ,N\right\}$ and $\left\{y_{t},t=1,2,\ldots ,N\right\}$, where N is the length of two time series, denote two random variables. Then, MHVGCA is performed as follows.

(1) Calculate two coarse-grained time series $X_{i}^{\left(s\right)},Y_{i}^{\left(s\right)}$ by averaging them at the same time scale s, in other words, calculate the first-order.

(1) $$\{\begin{array}{c} X_{i}^{\left(s\right)}=\frac{1}{s}\sum_{j=1}^{s}x_{(i-1)s+j}\\ Y_{i}^{\left(s\right)}=\frac{1}{s}\sum_{j=1}^{s}y_{(i-1)s+j} \end{array}$$

where s represents the scale factor and takes integer values 1,2,…,k(k≤N) and i ranges from 1 to $\left[\frac{N}{s}\right]$. The length on the coarse-grained time series is equal to the integer part of the length of the original time series divided by the scale factor s.

(2) Map the coarse-grained time series ${\left\{X_{i}^{\left(s\right)}\right\}}_{i=1}^{\left[\frac{N}{s}\right]}$ and ${\left\{Y_{i}^{\left(s\right)}\right\}}_{i=1}^{\left[\frac{N}{s}\right]}$ into a HVG.

(3) Compute the node degrees k. The degree $k_{i}$ of the *i*-th node gives the number of other nodes horizontally visible to this node,

(2) ki=∑j=1,j≠i⌊NS⌋Hi,j

where *H_i,j_* denotes whether or not two nodes *i* and *j* are horizontally visible. *H_i,j_* is set to 1, if all points $X_{l}^{\left(s\right)}$ between $X_{i}^{\left(s\right)}$ and $X_{j}^{\left(s\right)}$ fulfill the condition [8],

(3) $$\begin{array}{cc} \{\begin{array}{c} X_{i}^{\left(s\right)}>X_{l}^{\left(s\right)}\\ X_{j}^{\left(s\right)}>X_{l}^{\left(s\right)} \end{array}, & \forall l\in (i,j) \end{array}$$

which indicates that the two nodes i and j are horizontally visible, otherwise, $H_{ij}=0$.

When i and j are nearest neighbors, two nodes are naturally visible, namely $H_{ij}=1$. Consequently, the smallest value of $k_{i}$ is 2, if and only if the node falls in a valley and its nearest neighbors are both larger than it (i≠1, $\left[\frac{N}{s}\right]$). The value of $k_{i}$ can be exceptionally 1 for the first or last node $X_{i}^{\left(s\right)}$, if the nearest neighbor of the node is larger than $X_{i}^{\left(s\right)}$ (i=1 or $\left[\frac{N}{s}\right]$). No direction between nodes is defined in the HVGs, therefore, $H_{ij}=H_{ji}$.

(4) For two coarse-grained time series ${\left\{X_{i}^{\left(s\right)}\right\}}_{i=1}^{\left[\frac{N}{s}\right]}$ and ${\left\{Y_{i}^{\left(s\right)}\right\}}_{i=1}^{\left[\frac{N}{s}\right]}$, we obtain two degree sequences $k_{i}^{\left\{X\right\}}$ and $k_{i}^{\left\{Y\right\}}$, which are discrete and ordinal [29].

(5) Measure the strength of association between the degree sequences using the Goodman and Kruskal’s gamma [30].

(4) $$G=\frac{N_{s}-N_{d}}{N_{s}+N_{d}}$$

where $N_{s}$ is the number of concordant pairs (i.e., $k_{i}^{\left\{X\right\}}<k_{j}^{\left\{X\right\}}$, $k_{i}^{\left\{Y\right\}}<k_{j}^{\left\{Y\right\}}$, or $k_{i}^{\left\{X\right\}}>k_{j}^{\left\{X\right\}}$, $k_{i}^{\left\{Y\right\}}>k_{j}^{\left\{Y\right\}}$) and $N_{d}$ is the number of discordant pairs (i.e., $k_{i}^{\left\{X\right\}}<k_{j}^{\left\{X\right\}}$, $k_{i}^{\left\{Y\right\}}>k_{j}^{\left\{Y\right\}}$, or $k_{i}^{\left\{X\right\}}>k_{j}^{\left\{X\right\}}$, $k_{i}^{\left\{Y\right\}}<k_{j}^{\left\{Y\right\}}$). G measures the strength of association of the cross tabulated data when both variables are measured at the ordinal level. The value of G ranges from −1 (100% negative association, or perfect inversion) to +1 (100% positive association, or perfect agreement). G≈0 indicates the absence of association. Considering that the tied values occurred quite frequently in the degree sequences, we also constructed a statistic to determine whether the cross-correlation exists or not, and the results were consistent with the values of our proposed method.

(6) Change the scale s and repeat steps 1–5.

Finally, we obtain a series of G(s) that quantifies the strength of the interaction between the time series over multiple time scales. For two time series, the 1st-order MHVGCA reduces to classical horizontal visibility graph correlation analysis (HVGCA) while s=1.

### 2.2. Second-Order Multiscale Horizontal Visibility Graph Correlation Analysis Method

The second-order moment is recognized as an important tool in time series analysis since it overcomes a well-known limitation of the mean value method (i.e., the lack of information about deviation in random variables). We calculated two coarse-grained time series $X_{i}^{\left(s\right)},Y_{i}^{\left(s\right)}$ by the second-order statistical moment at the same time scale s (i.e., variance) based on the multiscale horizontal visibility graph correlation analysis.

(5) $$\{\begin{array}{c} X_{i}^{\left(s\right)}=\frac{1}{s}\sum_{j=1}^{s}{\left(x_{(i-1)s+j}-\langle x\rangle \right)}^{2}\\ Y_{i}^{\left(s\right)}=\frac{1}{s}\sum_{j=1}^{s}{\left(y_{(i-1)s+j}-\langle y\rangle \right)}^{2} \end{array}$$

where 〈x〉, 〈y〉 are the means over $\left\{x_{t}\right\}$ and $\left\{y_{t}\right\}$ just in the respective time window s.

**Figure 1:**
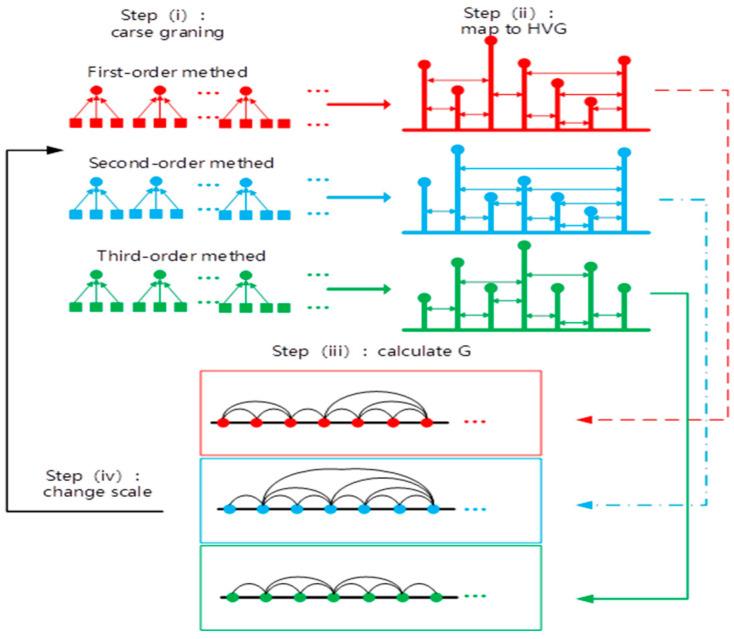
Schematic sketch of the higher-order multiscale horizontal visibility graph correlation analysis.

The procedure of the second-order MHVGCA is schematically illustrated in Figure 1.

In order to quantify the strength of the interaction between the time series over multiple time scales, we repeated steps 2–6 to obtain a series of G(s), named the second-order multiscale horizontal visibility graph correlation analysis (2nd-order MHVGCA). For the sake of convenience, the initial MHVGCA is called the first-order multiscale horizontal visibility graph correlation analysis (1st-order MHVGCA).

### 2.3. Third-Order Multiscale Horizontal Visibility Graph Correlation Analysis Method

Since skewness is related to extreme variations, it is also important for time series analysis. Our framework proposes a multiscale horizontal visibility graph correlation analysis of the third-order moment for the coarse-grained time series. We computed the third-order moment for overcoming the limitation of the 2nd-order method as follows,

(6) $$\{\begin{array}{c} X_{i}^{\left(s\right)}=\frac{1}{s}\sum_{j=1}^{s}{\left(x_{(i-1)s+j}-\langle x\rangle \right)}^{3}\\ Y_{i}^{\left(s\right)}=\frac{1}{s}\sum_{j=1}^{s}{\left(y_{(i-1)s+j}-\langle y\rangle \right)}^{3} \end{array}$$

where 〈x〉, 〈y〉 are the means over $\left\{x_{t}\right\}$ and $\left\{y_{t}\right\}$ just in the respective time window s.

Then, we repeated steps 2–6 to obtain a series of G(s), named the third-order multiscale horizontal visibility graph correlation analysis (3^rd^-order MHVGCA), for quantifying the strength of the interaction between time series.

To even further account for non-Gaussianity of random variables (including kurtosis), we defined the *n*^th^-order multiscale horizontal visibility graph correlation analysis (*n*^th^-order MHVGCA) as follows,

(7) $$\{\begin{array}{c} X_{i}^{\left(s\right)}=\frac{1}{s}\sum_{j=1}^{s}{\left(x_{(i-1)s+j}-\langle x\rangle \right)}^{n}\\ Y_{i}^{\left(s\right)}=\frac{1}{s}\sum_{j=1}^{s}{\left(y_{(i-1)s+j}-\langle y\rangle \right)}^{n} \end{array}$$

where 〈x〉, 〈y〉 are the means over $\left\{x_{t}\right\}$ and $\left\{y_{t}\right\}$ just in the respective time window s. Next, we repeated steps 2–6 to obtain a series of G(s).

## 3. Higher-Order MHVGCA for Artificial Time Series

The auto-regressive fractionally integrated moving average (ARFIMA) process can be modeled [31,32,33] to generate artificial time series with power-law auto-correlation. The generation procedure of an artificial series is defined as

(8) $$u_{i}=\sum_{j=1}^{\infty }a_{j}(\rho )y_{i-j}+\eta_{i}$$

where 0<ρ<0.5 is a free parameter; $a_{j}(\rho )=\frac{\Gamma (j-\rho )}{\Gamma (-\rho )\Gamma (i+j)}$ are weights; Γ(j) is the Gamma function; and $\eta_{i}$ is an independent and identically distributed (i.i.d.) Gaussian variable. By changing the parameter ρ, the method can efficiently generate signals with power-law correlation. The parameter ρ is related to the Hurst exponent H=0.5+ρ [28,34].

In our analysis, to account for the association between two variables, we employed the two-component auto-regressive fractionally integrated moving average stochastic process to generate two artificial variables with power-law cross-correlation.

(9) $$\{\begin{array}{c} u_{i}=\left[WU_{i}+(1-W)V_{i}\right]+\varepsilon_{i}\\ v_{i}=\left[WV_{i}+(1-W)U_{i}\right]+{\tilde{\varepsilon }}_{i}\\ U_{i}=\sum_{j=1}^{\infty }a_{j}(\rho_{1})u_{i-j}\\ V_{i}=\sum_{j=1}^{\infty }a_{j}(\rho_{2})v_{i-j} \end{array}$$

where $a_{j}(\rho )=\frac{\Gamma (j-\rho )}{\Gamma (-\rho )\Gamma (i+j)}$ are the weights used in Equation (8) with scale parameters $0<\rho_{1},\;\rho_{2}<0.5$; *W* is a free parameter to control the coupling strength between $\left\{u_{i}\right\}$ and $\left\{v_{i}\right\}$ (0.5 ≤ *W* ≤ 1); and $\varepsilon_{i}$ and ${\tilde{\varepsilon }}_{i}$ are independent and identically distributed (i.i.d) Gaussian variables with $\langle \varepsilon_{i}\rangle =\langle {\tilde{\varepsilon }}_{i}\rangle =0$ and $\langle \varepsilon_{i}{}^{2}\rangle =\langle {\tilde{\varepsilon }}_{i}{}^{2}\rangle =1$ [31,32]. For different values of *W*, the coupling strength between the variables $\left\{u_{i}\right\}$ and $\left\{v_{i}\right\}$ is 1 − *W*. Specifically, the process defined in Equation (9) reduces to two uncoupled cases and the cross-correlations vanish, when *W* = 1.

In this section, the two-component ARFIMA series $\left\{u_{i}\right\}$ and $\left\{v_{i}\right\}\left(i=1,2,\ldots ,N,N=30000\right)$ with parameters $\rho_{1}=\rho_{2}=0.3$ and *W* = 0.5, denoted by (ρ,W)=(0.3,0.5), were employed to detect the interactions between two time series. Large amounts of data will help lessen the boundary effects on the degree property [35,36]. Therefore, the maximum scale s=20 (the minimum length of the coarse-grained time series is 1500) was used here. For comparison, we then generated three pairs of time series: (ρ,W)=(0.3,0.5), (ρ,W)=(0.3,0.7), and (ρ,W)=(0.3,0.9). We applied the 1st-order MHVGCA method and 2nd-order MHVGCA to the artificial series. Preliminary results obtained with the two-component ARFIMA series suggest that G(s) values of the 2nd-order MHVGCA method are lower than the corresponding G(s) results of the 1st-order MHVGCA method.

**Figure 2:**
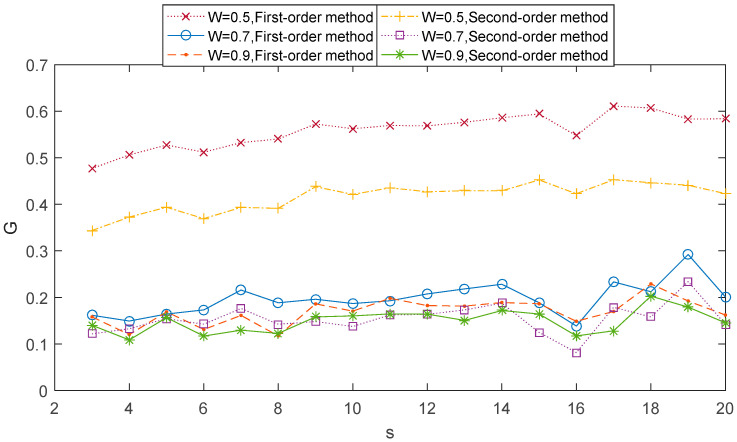
1st-order MHVGCA method and 2nd-order MHVGCA for the two-component auto-regressive fractionally integrated moving average process with $\rho_{1}=\rho_{2}=0.3$ and *W* = 0.5, 0.7 & 0.9.

We can deal with the two-component ARFIMA process through 1st-order MHVGCA and 2nd-order MHVGCA. The comparative relation between the two methods is shown in Figure 2, which includes three cases. For comparison, for the classical HVGCA G=0.48 was calculated for the case (ρ,W)=(0.3,0.5) first. For the sake of convenience, we defined the averaged G(s) values as $G^{\left(n\right)}=\langle G(s)\rangle$ for the *n*th-order MHVGCA. In the first case (ρ,W)=(0.3,0.5), the G(s) values of the 1st-order MHVGCA method fluctuated around the mean value of $G_{\left(0.3,0.5\right)}^{\left(1\right)}=0.56$, which is close to the classical HVGCA association G.

Due to the coarse-grained series of the signal that varies at each scale *s*, there exist small fluctuations in the association measure *G*(*s*). These small fluctuations can be deemed as clear evidence that shows that the variability of association at each time is instrumental in our comprehension of a more complex evolution dynamics.

The *G*(*s*) values of the 2nd-order MHVGCA method fluctuated around the mean value $G_{\left(0.3,0.5\right)}^{\left(2\right)}=0.41$, which indicates the continuous information exchange in variance. The result $G_{\left(0.3,0.5\right)}^{\left(1\right)}-G_{\left(0.3,0.5\right)}^{\left(2\right)}=0.15$ reflects the change between the mean and variance.

In the second case (ρ,W)=(0.3,0.7), the mean value $G_{\left(0.3,0.7\right)}^{\left(1\right)}>G_{\left(0.3,0.7\right)}^{\left(2\right)}$, and $G_{\left(0.3,0.7\right)}^{\left(1\right)}-G_{\left(0.3,0.7\right)}^{\left(2\right)}=0.05$. In the third case (ρ,W)=(0.3,0.9), the mean value $G_{\left(0.3,0.9\right)}^{\left(1\right)}\approx G_{\left(0.3,0.9\right)}^{\left(2\right)}$.

In the three cases, the mean value $G_{\left(0.3,0.5\right)}^{\left(1\right)}>G_{\left(0.3,0.7\right)}^{\left(1\right)}>G_{\left(0.3,0.9\right)}^{\left(1\right)}$, and $G_{\left(0.3,0.5\right)}^{\left(2\right)}>G_{\left(0.3,0.7\right)}^{\left(2\right)}>G_{\left(0.3,0.9\right)}^{\left(2\right)}$ are consistent with those in [31].

**Figure 3:**
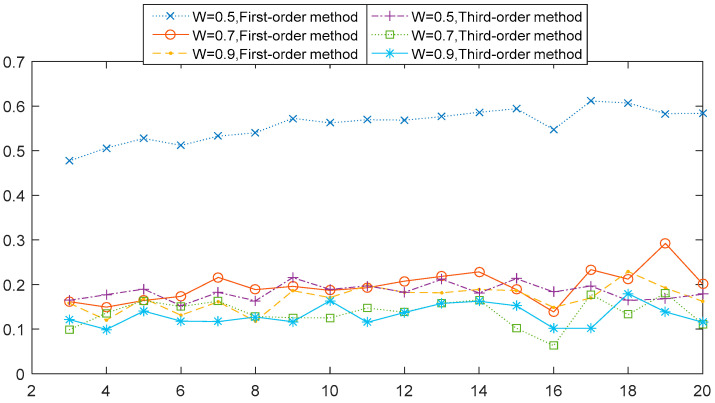
1st-order MHVGCA method and 3rd-order MHVGCA for the two-component ARFIMA process with $\rho_{1}=\rho_{2}=0.3$ and *W* = 0.5, 0.7, and 0.9.

The next observation concerns the comparative relation 1st-order MHVGCA vs. the 3rd-order MHVGCA. The numerically obtained values of G(s) for four cases are illustrated respectively in Figure 3. In the first case (ρ,W)=(0.3,0.5), the G(s) values of the 3rd-order MHVGCA method fluctuated around the mean value $G_{\left(0.3,0.5\right)}^{\left(3\right)}=0.18$, and $G_{\left(0.3,0.5\right)}^{\left(1\right)}>G_{\left(0.3,0.5\right)}^{\left(2\right)}>G_{\left(0.3,0.5\right)}^{\left(3\right)}$.

Table 1 tabulates the numerical values and their mean $G^{\left(n\right)}$ and standard deviation σ(G) for the cases regarding the 1st-order MHVGCA, 2nd-order MHVGCA, and 3rd-order MHVGCA method. There is a slight oscillation of the associations G(s) between the two variables, which indicates the persistent information exchange about the trend of average, standard deviation variance, and skewness on different scale s.

In the other cases of (ρ,W)=(0.3,0.7) and (ρ,W)=(0.3,0.9), the mean value *G* of the 1st-order MHVGCA was roughly the same as the mean value *G* of the 3rd-order MHVGCA that can reflect related information in skewness.

**Figure 4:**
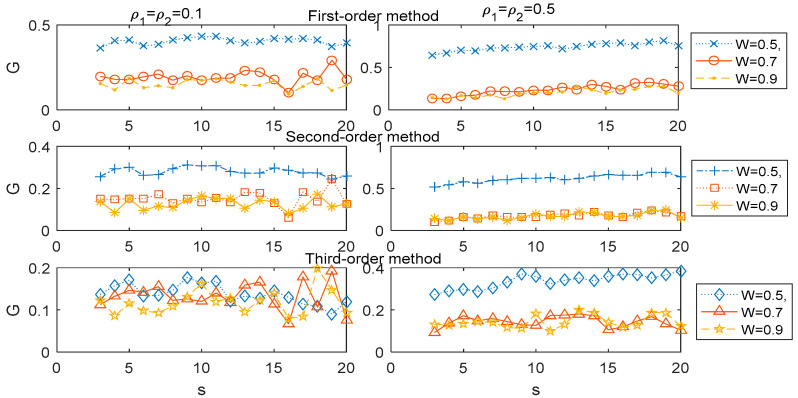
1st-order MHVGCA method, 2nd-order MHVGCA, and 3rd-order MHVGCA for the two-component ARFIMA process with $\rho_{1}=\rho_{2}=0.1$ and *W* = 0.5, 0.7 & 0.9 (**left**), and with $\rho_{1}=\rho_{2}=0.5$ and *W* = 0.5, 0.7 (**right**).

We now analyzed the association *G*(*s*) for the higher-order MHVGCA from the other six two-component ARFIMA cases through numerical experiments. In Figure 4, we illustrate the comparative relation of the 1st-order MHVGCA, 2nd-order MHVGCA, and 3rd-order MHVGCA for six two-component ARFIMA cases (ρ,W)=(0.1,0.5), (ρ,W)=(0.1,0.7), (ρ,W)=(0.1,0.9), (ρ,W)=(0.5,0.5), (ρ,W)=(0.5,0.7), and (ρ,W)=(0.5,0.9), respectively.

It can be seen that the association *G* gradually decreased as the free parameter *W* increased for fixed parameter ρ, scale *s*, and 1st-order MHVGCA, which is in excellent agreement with the results presented in [31]. For the 3rd-order MHVGCA, the association *G*(*s*) is based on the skewness of the signal and the gradual downward trend of *G*(*s*) was not pronounced as the free parameter *W* increased, especially for the case $\rho_{1}=\rho_{2}=0.1$.

## 4. Application of Higher-Order MHVGCA

### 4.1. Results and Analysis for Stock Time Series

We applied the higher-order MHVGCA method to the stock time series and unveiled the characteristics and correlations of different stock time series. For analyzing the features of financial markets, we selected the daily closing index values from two classical stock markets, the Shanghai Stock Exchange Composite Index (SSEC) and the Standard & Poor 500 Composite Stock Price Index (S&P500). Datasets were from January 04, 1993 to January 03, 2019.

**Figure 5:**
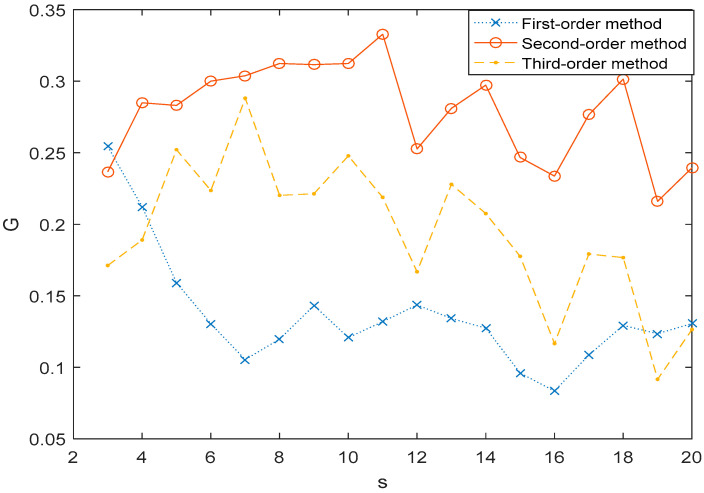
The association *G*(*s*) of the Stock Exchange Composite Index (SSEC) vs. Standard & Poors500 Composite Stock Price Index (S&P500) time series for 1st-order MHVGCA method, 2nd-order MHVGCA, and 3rd-order MHVGCA.

In Figure 5, we plotted the behavior of these series, with the association *G*(*s*) of MHVGCAs as functions of the scale s. The G(s) values of the 1^st^-order MHVGCA method fluctuated around the mean value $G_{\mathrm{SSEC}\text{-}S\text{\&}P500}^{\left(1\right)}=0.14$. The G(s) values of the higher-order MHVGCA method fluctuated around the mean values of $G_{\mathrm{SSEC}\text{-}S\text{\&}P500}^{\left(2\right)}$ and $G_{\mathrm{SSEC}\text{-}S\text{\&}P500}^{\left(3\right)}$. The mean values $G_{\mathrm{SSEC}\text{-}S\text{\&}P500}^{\left(1\right)}<G_{\mathrm{SSEC}\text{-}S\text{\&}P500}^{\left(3\right)}<G_{\mathrm{SSEC}\text{-}S\text{\&}P500}^{\left(2\right)}$ indicate the continued variations in skewness and variance. The results differed from the previous stochastic process example, which show that the applications of variance cause the relationship to strengthen in different stock time series. Consequently, variations in variance and skewness may provide more information to promote our understanding of the stock time series.

In order to further analyze the characteristics and correlations of different stock time series, we next selected the daily closing index values from 11 classical stock markets including the Shanghai Stock Exchange Composite Index (SSEC), Shenzhen Component Index (SZSE), Russian Trading System Index (RTS), Hang Seng Index (HSI), Tokyo Nikkei-225 Index (TN225), Deutscher Aktienindex (DAX), Cotation Assistée en Continu 40 (CAC40), Standard & Poors500 Composite Stock Price Index (S&P500), National Association of Securities Dealers Automated Quotations (NASDAQ), Dow Jones Industrial Average (DJI), and Brazil’s Bovespa Stock Index (IBOVESPA).

**Figure 6:**
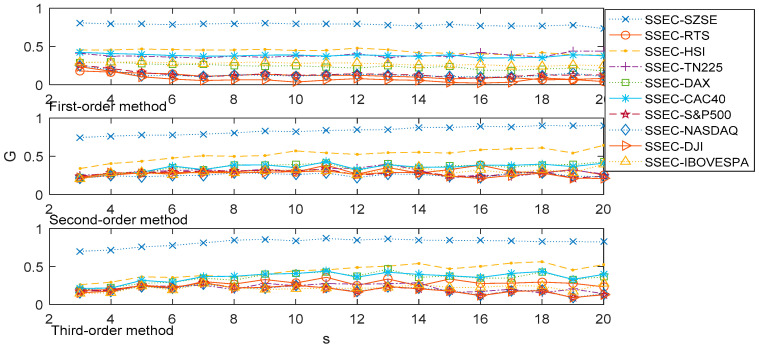
The association *G*(*s*) of the SSEC vs. other 10 stock time series for the 1st-order MHVGCA method, 2nd-order MHVGCA, and 3rd-order MHVGCA.

Concerning the association of stock indices, we demonstrated the associations between the SSEC and the other 10 stock indexes on multiple time scales by the 1st-order, 2nd-order, and 3rd-order MHVGCA in Figure 6.

The association between the SSEC and SZSE performed relevantly different from the other associations. For the SSEC and SZSE, the association *G*(*s*) between the two variables fluctuated about the mean value of 0.78 for the 1st-order MHVGCA. This indicates the close information exchange between the SSEC and SZSE. For the association *G*(*s*) between the SSEC and American stock markets (DJI, NASDAQ, and S&P500) all decreased progressively with increasing scale, from 0.25 to roughly 0.1 for the 1st-order MHVGCA. This indicates that the SSEC has less of a relationship with American stock markets. The European stock markets are in an intermediate state between the Chinese markets and American markets, where the associations between the SSEC and European stock markets are hardly relevant.

For higher-order MHVGCA, the association *G*(*s*) between the SSEC and SZSE gradually increased with increasing scale, from 0.75 to roughly 0.90 for the 2nd-order MHVGCA, and fluctuated with increasing scale from 0.76 to roughly 0.87 for the 3rd-order MHVGCA. The results indicate that the associations between the SSEC and SZSE are relevant. The association G(s) between the SSEC and American stock markets all fluctuated with increasing scale from 0.21 to roughly 0.32 for the 2nd-order MHVGCA, and fluctuated with increasing scale from 0.09 to roughly 0.25 for the 3rd-order MHVGCA. These results indicate that the SSEC has less of a relationship with American stock markets. And are consistent with our results from the 1st-order MHVGCA.

It can be seen that the largest association measure *G*(*s*) were obtained by the SSEC and SZSE for the 1st-order, 2nd-order, and 3rd-order MHVGCA, which show that there is a close association between these two Chinese mainland stock markets. The next largest *G*(*s*) were acquired by the SSEC and HSI to demonstrate the strong correlations between the Chinese stock markets, especially in the results of the 2nd-order MHVGCA, where the associations between the SSEC and HSI gradually increased with increasing scale from 0.34 to 0.64. This indicates the information exchange between the Chinese stock markets.

### 4.2. Results and Analysis for Aero-Engine Time Series

In this section, we applied the higher-order MHVGCA method to the aero-engine time series and exposed the associations of different aero-engine performance parameter time series. To analyze the features of the performance parameters, the study started with an investigation of the association between the low-spool rotor speed (N1) and high-spool rotor speed (N2).

**Figure 7:**
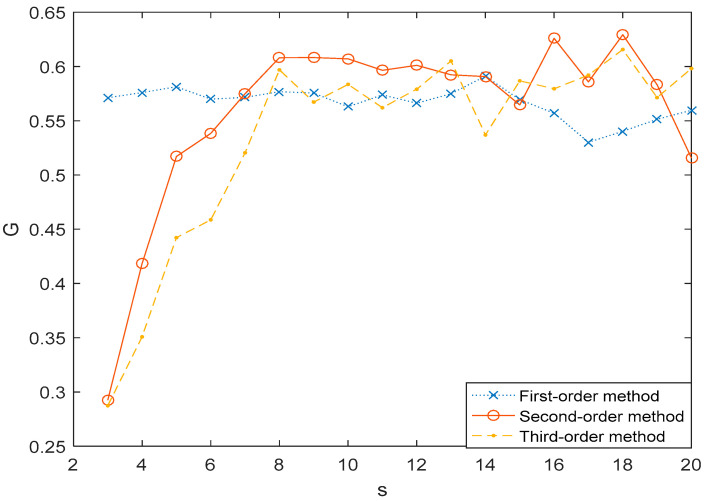
The association *G*(*s*) of N1 vs. N2 time series for 1st-order MHVGCA method, 2nd-order MHVGCA, and 3rd-order MHVGCA.

In Figure 7, we plotted the association *G*(*s*) of MHVGCA as a function of the scale s for the 1st-order, 2nd-order, and 3rd-order MHVGCA methods. The G(s) values of the 1st-order MHVGCA method fluctuated around the mean value of $G_{N1\text{-}N2}^{\left(1\right)}=0.56$. The results indicate that the associations between the N1 and N2 are relevant and a similar phenomenon was also observed in [37,38]. The G(s) values of the higher-order MHVGCA methods fluctuated around the mean value of $G_{N1\text{-}N2}^{\left(2\right)}=0.59$, $G_{N1\text{-}N2}^{\left(3\right)}=0.58$ when s>7. For s>7, the mean values $G_{N1\text{-}N2}^{\left(1\right)}<G_{N1\text{-}N2}^{\left(3\right)}<G_{N1\text{-}N2}^{\left(2\right)}$ indicate the continued variations in skewness and variance.

Aero-engine behavior can be described by aero-engine gas path parameters and their temporal and qualitative relationships. Parameter N1 was chosen to indicate the thrust by GE and Rolls-Royce, which indicate that N1 is the most important gas path variable. Parameter N2 is a spare parameter for N1 to indicate the thrust of the aero-engine. When s>7, G>0.5. This result indicates constant positive information exchange between the N1 and N2, which had excellent agreement with our expectations.

**Figure 8:**
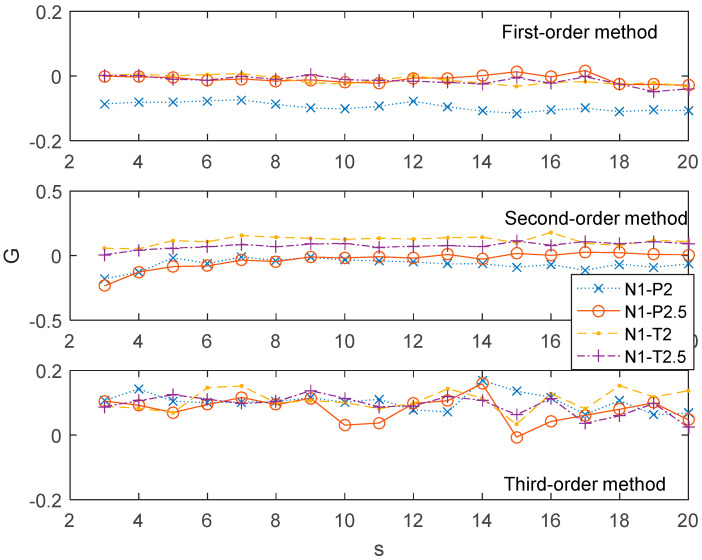
The association *G*(*s*) of N1 vs. the other four time series for the 1st-order MHVGCA method, 2nd-order MHVGCA, and 3rd-order MHVGCA.

In order to investigate the associations of different parameter series, we selected four performance parameters including the pressure subsystem parameters (P2 and P2.5) and the temperature subsystem parameters (T2 and T2.5). P2 and P2.5 are the abbreviations for total pressure of the compressor inlet at station 2 and station 2.5, respectively. Here, station means a vertical reference plane along the fuselage for measurements. The ratio of P2.5/P2 was used to estimate the performance of the low pressure compressor. T2 and T2.5, the abbreviations of the temperature of compressor inlet at station 2 and station 2.5, are concurrent with the P2 and P2.5. The associations between the N1 and the other four performance parameters series on multiple time scales by the 1st-order, 2nd-order, and 3rd-order MHVGCA are demonstrated in Figure 8.

The *G*(*s*) values between the N1 series and the pressure subsystem parameters (P2 and P2.5) and the temperature subsystem parameters (T2 and T2.5) were roughly equal 0, where the associations between the N1 series and the pressure subsystem parameters and the temperature subsystem parameters were hardly relevant. Specifically, a negative relationship G(s)<−0.1 between N1 and P2 was shown for scales s>14. The *G*(*s*) values fluctuated around zero, which indicate the absence of an association between N1 and the three other parameters. For the 2nd-order MHVGCA method, the subtle negative relationship between the N1 and the pressure subsystem parameters (P2 and P2.5) was regained. For the 3rd-order MHVGCA method, all curves of the *G*(*s*) values between the N1 series and the pressure subsystem parameters and the temperature subsystem parameters were tangled together. These results indicate the delicate information exchange between the skewness of N1 and the four other parameters. In contrast, the positive information exchange was hidden by the average and variance.

## 5. Conclusions

In this paper, we proposed a novel method for deriving interactions between non-stationary time series through higher-order statistics of HVG. We compared these new methods with classical measures by applying them to correlated a two-component ARFIMA stochastic time series. We then applied the higher-order MHVGCA to stock markets and aero-engine performance parameters, and measured the interactions in dynamic systems.

For the stock time series, our results indicate that concerning closing index values, there exists little information exchange between the Chinese stock markets and the American–European stock markets, whereas the SSEC and SZSE, by the 1st-order MHVGCA method, 2nd-order MHVGCA, and 3rd-order MHVGCA, show frequent and abundant information exchange in the Chinese domestic stock markets. Consequently, that the information exchange between the SSEC and HSI gradually increases with increasing scale is revealed by the 2nd-order MHVGCA and 3rd-order MHVGCA.

For the aero-engine performance parameters, our results showed that there is little information exchange between the engine rotor subsystem and the pressure–temperature subsystem. However, the close information exchange between N1 and N2 within the engine rotor subsystem is demonstrated by the 1st-order MHVGCA method, 2nd-order MHVGCA, and 3rd-order MHVGCA.

## Figures and Tables

**Table 1:** 1st-order MHVGCA, 2nd-order MHVGCA, and 3rd-order MHVGCA and their mean $G^{\left(n\right)}$ and standard deviation σ(G) for the two-component ARFIMA process with $\rho_{1}=\rho_{2}=0.3$ and *W* = 0.5.

	G(3)	G(4)	G(5)	G(6)	G(7)	G(8)	G(9)	G(10)	G(11)	G(12)
1st-order	0.48	0.51	0.53	0.51	0.53	0.54	0.57	0.57	0.57	0.57
2nd-order	0.16	0.15	0.16	0.17	0.22	0.19	0.20	0.19	0.19	0.21
3rd-order	0.16	0.12	0.17	0.13	0.16	0.12	0.19	0.17	0.20	0.18
	**G(13)**	**G(14)**	**G(15)**	**G(16)**	**G(17)**	**G(18)**	**G(19)**	**G(20)**	$G^{\left(n\right)}$	σ(G)
1st-order	0.58	0.59	0.59	0.55	0.61	0.61	0.58	0.58	0.56	0.04
2nd-order	0.22	0.23	0.19	0.14	0.23	0.21	0.29	0.20	0.20	0.04
3rd-order	0.18	0.19	0.19	0.15	0.17	0.23	0.19	0.16	0.17	0.03
